# How Can Model Comparison Help Improving Species Distribution Models?

**DOI:** 10.1371/journal.pone.0068823

**Published:** 2013-07-09

**Authors:** Emmanuel Stephan Gritti, Cédric Gaucherel, Maria-Veronica Crespo-Perez, Isabelle Chuine

**Affiliations:** 1 CEFE, UMR 5175 CNRS/Université Montpellier II, 1919, Route de Mende, 34293, Montpellier, France; 2 UMR System, Montpellier SupAgro, 2 place Viala, Bât. 27, 34060 Montpellier cedex 1, France; 3 UMR AMAP-INRA, Montpellier, France; 4 French Institute of Pondicherry, IFP-CNRS, Pondicherry, India; DOE Pacific Northwest National Laboratory, United States of America

## Abstract

Today, more than ever, robust projections of potential species range shifts are needed to anticipate and mitigate the impacts of climate change on biodiversity and ecosystem services. Such projections are so far provided almost exclusively by correlative species distribution models (correlative SDMs). However, concerns regarding the reliability of their predictive power are growing and several authors call for the development of process-based SDMs. Still, each of these methods presents strengths and weakness which have to be estimated if they are to be reliably used by decision makers. In this study we compare projections of three different SDMs (STASH, LPJ and PHENOFIT) that lie in the continuum between correlative models and process-based models for the current distribution of three major European tree species, *Fagus* *sylvatica* L.*,* *Quercus* *robur* L. and *Pinus* *sylvestris* L. We compare the consistency of the model simulations using an innovative comparison map profile method, integrating local and multi-scale comparisons. The three models simulate relatively accurately the current distribution of the three species. The process-based model performs almost as well as the correlative model, although parameters of the former are not fitted to the observed species distributions. According to our simulations, species range limits are triggered, at the European scale, by establishment and survival through processes primarily related to phenology and resistance to abiotic stress rather than to growth efficiency. The accuracy of projections of the hybrid and process-based model could however be improved by integrating a more realistic representation of the species resistance to water stress for instance, advocating for pursuing efforts to understand and formulate explicitly the impact of climatic conditions and variations on these processes.

## Introduction

Recent climatic and atmospheric CO_2_ concentration changes have been shown to cause modifications in ecosystems distribution, structure and function [1,2]. These modifications greatly alter ecosystems biodiversity, distribution and ecosystems services leading to socioeconomic and financial costs [3,4]. However, in several cases the loss of biodiversity and associated services could be avoided or minimised by developing adaptive management strategies [3] supported by intelligible species’ potential responses to climate change integrated indicators. These indicators are often provided by species distribution models (SDMs) [5]. Yet, forecast of species’ distributions presents substantial discrepancies according to the type of predictive modelling approach used [2,6–8] highlighting the uncertainties associated with these predictions [6,9].

These uncertainties may puzzle environmental decision makers and shade doubt on the credibility of species’ distribution projections. Therefore, rigorous estimations of models strengths, weakness and discrepancies have to be performed. In this regard, we present here the comparison of different kinds of SDMs simulating the current distributions of three major European forest tree species. We identify the reasons for the discrepancies observed in the simulations in order to propose new research avenues in the development and amelioration of such models.

One fundamental assumption in plant biogeography is that at a continental scale, a potential species’ distribution is mainly determined by climatic and environmental conditions [10]. Seminal formalised ideas about the relationships between environmental factors and species’ distribution emerged with the niche concept by Grinell [11], defined as the set of environmental conditions required by species to attain positive population growth rate. Albeit the term “ecological niche” has been used and defined in numerous ways since then [12], the Hutchinsonian [13] formalisation of the niche as a multi-dimensional space of suitable environmental conditions is still of prime importance in ecological modelling studies [14].

Indeed, the definition of the realised niche being a subset of the fundamental niche corresponding to a favourable combination of environmental variables at a given time and location for a species’ occurrence [15], remains the essential assumption of correlative SDMs. These models rely on the definition of species-specific bioclimatic envelopes based on a set of bioclimatic limits constraining the observed geographical species expansion. These limits are then superimposed on the geographical distribution of the selected bioclimatic variables for a given scenario [16]. Such models have the advantages of allowing rapid analyses for numerous individual species even when the expansion limiting factors are poorly known [16].

However, a number of methodological issues regarding the use of this type of model have been raised (for a review, see [17]), among them being:

• The statistical techniques used to construct models and the selection of explanatory variables included are responsible for the largest discrepancy in projections [18].
• Many non-climatic factors may influence species distributions such as migration rates, landscape continuity or biotic interactions [19].
• The key assumption of equilibrium between species distribution and environmental conditions may never be verified [20,21].

Recently, process-based SDMs have been developed which are deeply grounded in the second definition of the niche proposed by Rosenzweig [22] as the set of adaptive traits allowing a species to survive in various environments. A few of them have been developed for plants [23–25] or animals [26,27] over the past decades. They incorporate the plasticity of responses for several key traits and processes governing species establishment, growth, survival and reproduction in response to environmental factors. The explicit formulation of functional relationships between environmental factors, species traits and biological processes makes the projections of this kind of models more credible and particularly under novel combination of climatic factors (ie. unseen combinations of temperature, precipitations and CO_2_ atmospheric’ concentrations) [2,6,28,29]. Therefore, we expect that they might be used more reliably to project the impact of environmental change in the coming centuries [26], increasing thus greatly their value for climate change mitigation and adaptation strategies [6].

However, the development of these models requires a lot of information on traits, and processes modelled, to produce pertinent sets of indicators for how vegetation will respond spatially and temporarily to environmental conditions’ modifications [30].

A third category of SDMs, called hybrid SDMs, emerged in the 1990s combining the correlative and the process-based approach to model the fundamental niche [27,30]. The first models of this kind which were developed are Biogeographical Equilibrium Models (BEMs; [31]) followed by Dynamic Vegetation Models (DGVMs [27]). These models were primarily designed to project biomes distributions and later plant functional type distributions. Yet, recently these models have been also used at the species level [32–34]. DGVMs, use bioclimatic limits (determined using biomes or species’ observed distributions) as purely correlative SDMs, but also simulate processes related to growth, with formulations parameterised using field or laboratory measurements like purely process-based SDMs.

Recent studies have compared the different kinds of SDMs. Morin & Thuiller [8], Kramer et al. [35] and Cheaib et al. [6] compared the ensemble of correlative models to process-based SDMs and hybrid SDMs under future climate scenarios. They found that although all models projected northward species distribution shifts, the amplitude of these shifts were increasingly divergent, depending on the climatic warming scenario.

Our objectives in the present study were first to assess and understand the consistency between conceptually different SDMs: a correlative model (STASH), a hybrid model (LPJ) and a process-based model (PHENOFIT). Using a present-day climatic dataset, each model is used to project current potential distributions of three common tree species *Fagus* *sylvatica* L., *Quercus* *robur* L. and *Pinus* *sylvestris* L. at the European scale. These species have been selected as being major components of European’s temperate and boreal forests [36], spanning broad environmental conditions and for which the models’ required data are available. Simulations are compared by determination of their agreement with the species observed distribution and by testing the consistency of simulations using the comparison map profile method (CMP) [37,38].

Second, we aimed to identify the strengths and weaknesses of each model. We assume that pinpointing areas and scales of divergence and agreement is a relevant way to distinguish necessary “processes” or traits to consider when projecting vegetation distributions. This should identify the fundamental processes to refine or implement in a model’s generation to address the shortcoming of the current one.

## Materials and Methods

### Species distribution models

#### STASH

STASH is a correlative envelope model based on physiological bioclimatic pertinent descriptors [39]. These descriptors are assumed to drive the species’ physiological responses to climate. Some of them act as on–off switches and limit the spatial distribution of the species, while others weight the degree of establishment success in a grid cell. Each parameter is fitted based on the species current distribution. For this reason, STASH is considered here as a correlative model although its parameters are predefined and not selected statistically as in most SDMs. See [39] and Appendix S1 & Table S1 in Supporting Information for further details and model parameterisation. Stash is available from the EMBERS group of Lund University, upon request.

#### LPJ

LPJ is a dynamic general vegetation model combining bioclimatic limits to the species establishment and survival and explicit description of mechanistic of ecosystems’ processes such as physiology, biochemistry, vegetation dynamics and carbon and water fluxes [27]. A minimum set of bioclimatic limits are used to define the spatial boundaries of the species’ distributions. Using climatic, soil and CO_2_, LPJ estimates growth-related indices such as leaf area index (LAI) or net primary production (NPP). Here, the version described in Gritti et al. [40] was used but did not take inter specific competition into account. The simulations were performed at the species level, using specific parameters when available, or the generic parameters of the corresponding plant functional type described in Smith et al. [41].

See [42] and Appendix S1 & Table S1 for further details and model parameterisation. LPJ is available from the EMBERS group of Lund University, upon request.

#### PHENOFIT

PHENOFIT is a process-based SDM describing tree species potential distributions. It estimates the fitness of an average individual of a species in response to climatic and environmental conditions. The model relies on the assumption that species adaptation to abiotic conditions is tightly related to its capacity to synchronise its annual life cycle with seasonal climatic variations, directly affecting its probability to survive and to reproduce. Thus, annual survival probability of the considered species is the product of i) its probability of surviving climatic stress (frost and drought) until the following reproductive season and ii) its probability of producing viable seeds before the end of its current annual cycle. The model has been validated for a dozen American tree species [8,23,43,44]. See Appendix S1 & Table S1 for full details and model parameterisation. PHENOFIT is available from the Bioflux group of CEFE/CNRS, upon request.

### Simulations

To simulate the three species distributions with the three SDMs, we used observed climate and atmospheric CO_2_ concentrations from the ATEAM project dataset (http://www.pik-postdam.de/ateam [45]). This dataset covers the European window with a 10' resolution, and contains monthly values of temperature, precipitation and percentage full sunshine.

Monthly data from the first thirty years were used repeatedly to run the LPJ from bare ground, with a spin up period of 500 years, until carbon pool equilibrium was attained. This equilibrium state was used as the starting point for the model which was driven using the full dataset for the period 1901-2000.

Monthly data were interpolated to daily values for the same period, following classical methods used by several weather generators (e.g. CLIGEN [46]) to drive PHENOFIT.

Monthly means were calculated over a twenty-year period (1981-2000) as input data for STASH. Supplementary simulations were conducted to disentangle the individual effects of bioclimatic limits and growth processes within LPJ species distribution projections by omitting the correlative component of the model (ie. no bioclimatic constraints). Indeed, four bioclimatic limits in LPJ drive species survival and establishment: minimum GDD5 for establishment (GDD5mine); minimum temperature of the coldest month for survival and establishment (respectively Tcoldmins and Tcoldmine); maximum temperature of the coldest month for establishment (Tcoldmaxe) (Appendix S1 & Table S1 [39]). These limits represent known or likely physiological limiting mechanisms defining the climate space in which a species may occur. The specific values are taken from the forestry literature [47] and by comparison of current species distributions with bioclimatic variables [34].

### Model evaluation and comparison

Model outputs were occurrence probability for STASH, LAI for LPJ and a fitness index for PHENOFIT. LAI and fitness are used as estimates for species occurrence probability. The three indexes were standardised as continuous variables ranking from 0 to 1 to ease comparison of model projections. To evaluate model projection accuracy, we computed the AUC (Area Under the Receiving Operating Curve (ROC; [48]) and the Cohen’s kappa statistic [49] using species observed distribution maps from Atlas Flora Europea [50] completed by Laurent et al. [51] (See Figure S1). The AUC [48] is used here as a single threshold-independent measure of model performance. AUC values range between 0.5 and 1, for which values > 0.7 indicate a good fit according to the guidelines of Swets [52]. To calculate Cohen’s kappa, we transformed model outputs into dichotomous presence-absence projections using a specific threshold calculated as the model’s output value leading to maximal distance between the ROC and the 1:1 curve. Kappa ranges between -1 and 1, for which values between 0.4 and 0.75 are usually considered as a good fit and values >0.75 an excellent fit according to Landis & Koch [53]. These two statistics give an overall estimation of the accuracy of model projections but do not allow a localisation of model weaknesses [37]. For this reason, we also used the Comparison Map Profile method (CMP; [37,38]) to detect spatial similarity and difference patterns, as well as their spatial resolution, at the European scale using three indices:

• The Cohen’s kappa (Kappa) to compare model fit with observed distributions. This index gives an integrated idea about the commission and omission modelled relatively to presence and absence of species. Note that the Kappa value computed through the CMP method is relatively low, when compared with monoscale spatial classical methods essentially due to spatial averaging.
• The absolute distance (D) between model projections. This index gives an idea about absolute differences between model outputs.
• The cross-correlation coefficient (CC) to compare spatial patterns between model projections. This index gives an idea about relative variations, such as similar or contrasting directions of gradients and common anisotropies, between model outputs.

The CMP method is based on a circular moving window comparison covering the entire considered window. In addition, the moving window process is repeated several times by increasing the window size from scale 1 (± 1 pixel around the central one, approximate window size: 0.5°x0.5°) to scale 20 (± 20 pixels around the central one, approximate window size: 8°x8°) (See Figure S2). Scale 1 resembles a pixel-by-pixel comparison of the two images, while scale 20 informs on large gradient similarities between the compared images. This approach permits us to identify the spatial scale at which similarities and differences between images appear. These similarities are gathered into successive monoscale maps. The index profile provides the averaged values of all moving windows computed at the same scale across the image for each monoscale. We also computed the multiscale index map as the average pixel by pixel of all its monoscale maps. Both the profile and the multiscale map are complementary giving scaling and spatial information about the image comparison, respectively. Low Kappa and CC values indicate that the similarity between original images is poor, whereas low D values indicate good agreement. Hence, the CMP method highlights different spatial patterns between observed and projected species presence and of projected scores. For detailed description of the CMP method see Gaucherel et al. [37] and the associated software (http://umramap.cirad.fr/amap2/logiciels_amap/index.php?page=cmp).

## Results

### Model validation

**Figure 1:**
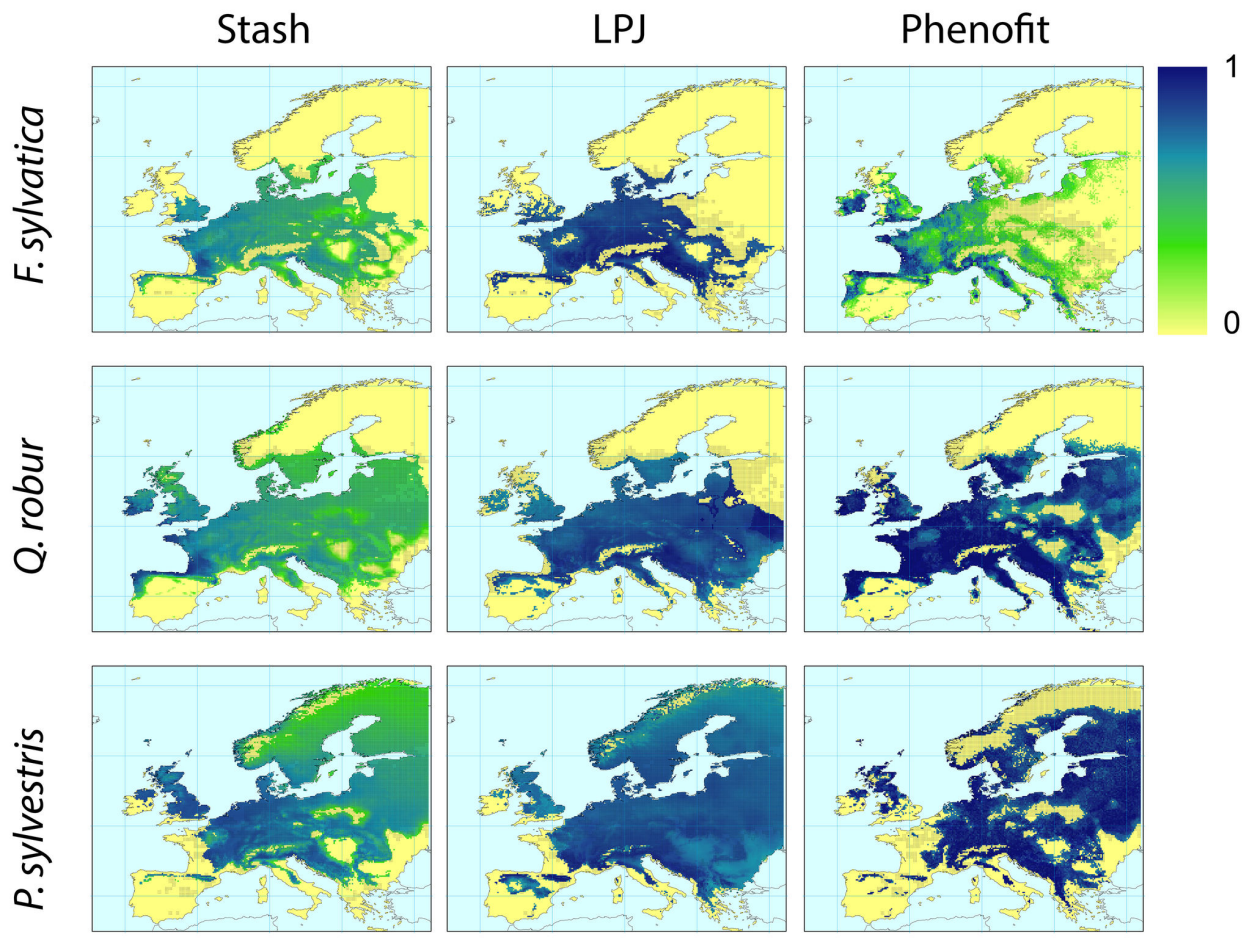
Projection of tree species current distributions by the three models after applying the species specific threshold (columns: STASH; LPJ; PHENOFIT; lines: *F* *. sylvatica* ; *Q* *. robur* ; *P* *. sylvestris* ; black dots: current observed distribution).

**Figure 2:**
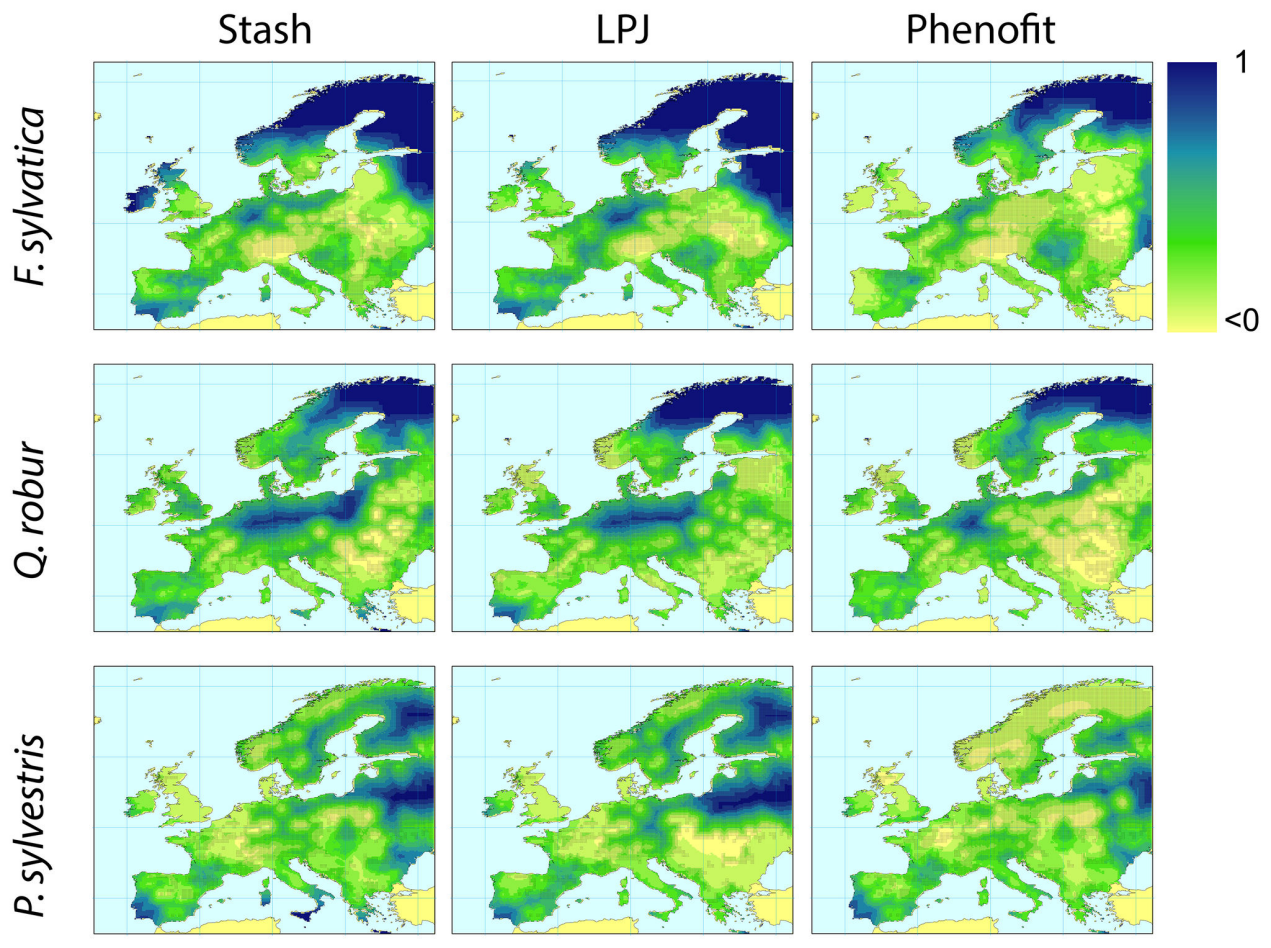
Average Kappa over the 20 monoscales for model projections of species present distributions (columns: STASH; LPJ; PHENOFIT; lines: *F* *. sylvatica* ; *Q* *. robur* ; *P* *. sylvestris* ; black dots: current observed distribution).).

According to the averaged AUC and Kappa values and maps of projected current distributions, model projections for the three species were accurate across the majority of Europe, for all models (Table 1 Figures 1 & 2). Model predictions were most reliable for *Q* *. robur* (except with LPJ) and least reliable for *P* *. sylvestris* . This suggests that the three models, despite being different, are able to capture the main climatic constraints for species distributions.

**Table 1:** Accuracy of projection of *F* *. sylvatica* *,* *Q* *. robur* *,* *P* *. sylvestris* present distributions by STASH, LPJ, and PHENOFIT (corresponding to Figure 1).

Species	Model	AUC	SPT	kappa_0_	Kappa_mean_
*F* *. sylvatica*	STASH	0.84	0.1	0.598	0.453
	LPJ	0.87	0.761	0.623	0.496
	PHENOFIT	0.78	0.138	0.438	0.324
*Q* *. robur*	STASH	0.85	0.005	0.630	0.396
	LPJ	0.82	0.670	0.529	0.374
	PHENOFIT	0.79	0.576	0.478	0.308
*P* *. sylvestris*	STASH	0.64	0.304	0.474	0.331
	LPJ	0.68	0.590	0.357	0.312
	PHENOFIT	0.68	0.704	0.321	0.228

kappa_0_ is the Kappa calculated for the monoscale 0 (pixel by pixel comparison) and Kappa_mean_ is the average Kappa calculated for the 20 monoscales (corresponding to the Figure 2). Species Presence Threshold (SPT) is defined as the inflexion point of the ROC curve and represents the specific threshold above which the focal species is considered as present in model projections.

Yet, discordances between projections and observations (Figures 1 & 2) are noticeable at specific locations. Visual comparisons between bioclimatic variables maps (See Figure S3) and additional LPJ simulations with no bioclimatic correlation constraints (data not shown) show that the Tcoldmins and Tcoldmine bioclimatic limits used in LPJ are responsible for the discrepancy between *F* *. sylvatica* and *Q* *. robur* north-eastern projected range limits, while water stress is responsible for the discrepancy between *Q* *. robur* and *P* *. sylvestris* south-eastern projected range limits. They also reveal that the poor explicit representation and parameterisation of the hydric constraint in the version of PHENOFIT used in this study is responsible for an overestimation of the effect of water stress on the eastern range limits of *F* *. sylvatica* and *Q* *. robur* . PHENOFIT underestimated the presence of *P* *. sylvestris* in its Scandinavian marginal distribution, probably due to local adaptation of Scandinavian population phenology that we could not incorporate in this study (due to a lack of phenological data for this region). *P* *. sylvestris* indeed exhibits strong adaptive differentiation among populations across its range [54].

The three models fail to reproduce the altitudinal distribution of *F* *. sylvatica* in the Alpine region, but this is mainly due to the coarse resolution of the observed current distribution data and the simple downscaling method used in this study. Low accuracy seems to occur also regarding the British Isles for *F* *. sylvatica* and *P* *. sylvestris* where Flora Europaea report their absence. However, more recent species distribution datasets report the presence of these species in the British Isles [55].

**Figure 3:**
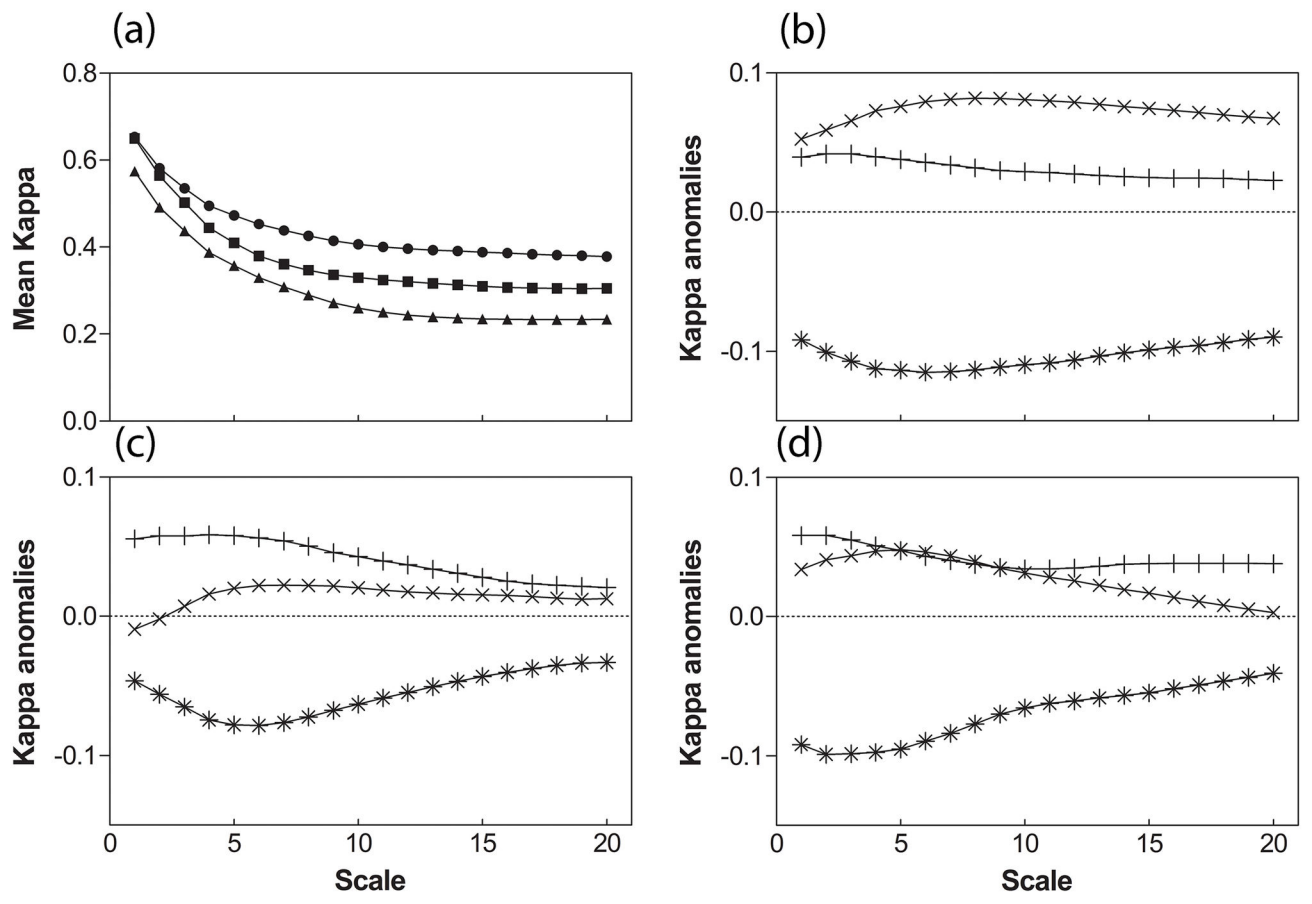
Mean Kappa of the 3 models projections for species present distributions ((a) circle: *F* *. sylvatica* ; square: *Q* *. robur* ; triangle: *P* *. sylvestris* ) and relative anomalies (×: LPJ; +: STASH; *: PHENOFIT) for (b) *F* *. sylvatica* ; (c): *Q* *. robur* ; (d): *P* *. sylvestris* .

Model projections are systematically more accurate (approximately two times better) at finer than at larger spatial scales (Figure 3a). This results from the fact that it is usually more difficult to match large gradients than local patterns, and that the three models describe the relationships between local climate and local species presence. At fine scales, the model may match the observation due to a correct handling of concerned processes or by chance (pure random patterns would lead to an averaged Kappa value around 0.01 at scales broader then 2). To maintain high Kappa values at broader scales, the model should match observations in a larger number of locations, with the correct spatial structure, which is far less probable.

The profiles of the Kappa anomalies exhibit minima, i.e. worse fits, for different scales according to the model. Generally, STASH presents minima at large scales when LPJ and PHENOFIT present minima at low scales (except in the case of *P* *. sylvestris* for LPJ) suggesting that process grounded models handle broad scales processes more efficiently. This is particularly striking in the case of PHENOFIT, which has lower Kappa values on average than STASH and LPJ (Figure 3 b, c & d). This reflects a stronger control of species distributions in PHENOFIT by temperature, a parameter that exhibits broad scale patterns. Phenology is indeed the keystone of this model and is strongly controlled by temperature. Interestingly, LPJ and STASH exhibit similar and higher relative Kappa anomalies profiles than PHENOFIT, suggesting that LPJ and STASH share common key features in their projections. These features are the bioclimatic limits, not derived from processes, driving species establishment, survival and accordingly distribution, in LPJ.

This latter result is confirmed by the additional simulations done with LPJ. They indeed show that whatever the species, the area within which NPP and LAI are positive, always encompasses the survival area driven by bioclimatic limits which itself always encompasses the establishment area also driven by bioclimatic limits. Survival and establishment reduced respectively the growth area by 66% and 59% for *Q* *. robur* , 52% and 48% for *F* *. sylvatica* , 88% and 76% for *P* *. sylvestris* . Distribution limits of *F* *. sylvatica* , *Q* *. robur* and *P* *. sylvestris* are thus to a very large extent driven by the bioclimatic limits determining survival and establishment and not by growth processes in LPJ. A part of the deviance from the observed distribution is thus directly due to these bioclimatic limits, as the comparison between model projections also suggested, in particular concerning the Tcoldmins and Tcoldmine bioclimatic limits.

### Model comparison

**Figure 4:**
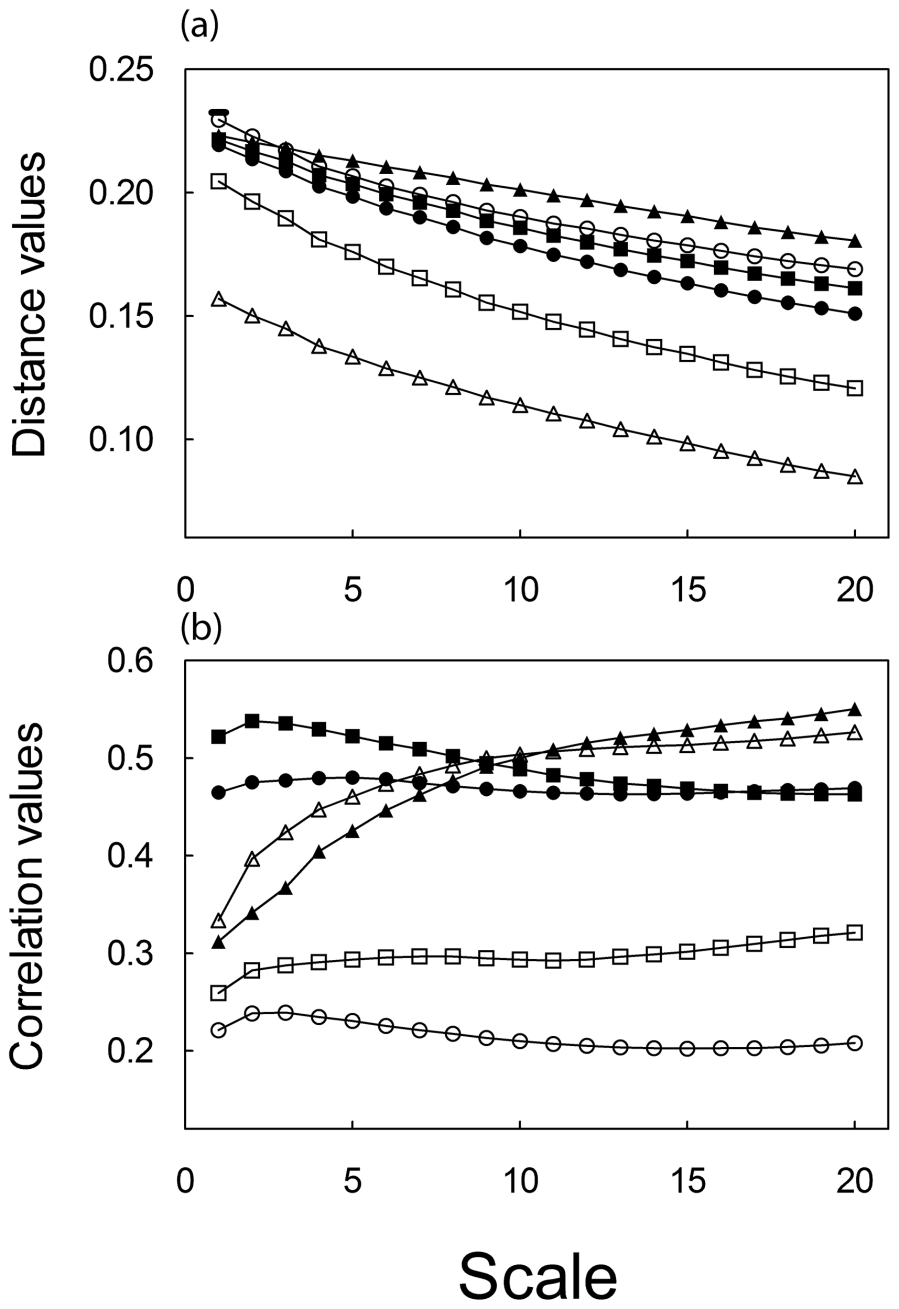
Mean absolute distance (a) and mean cross-correlation coefficient (b) between models and associated standard deviation. Circle: *F* *. sylvatica* ; Square: *Q* *. robur* ; Triangle: *P* *. sylvestris* . Black: LPJ-STASH; Open: LPJ-PHENOFIT.

**Figure 5:**
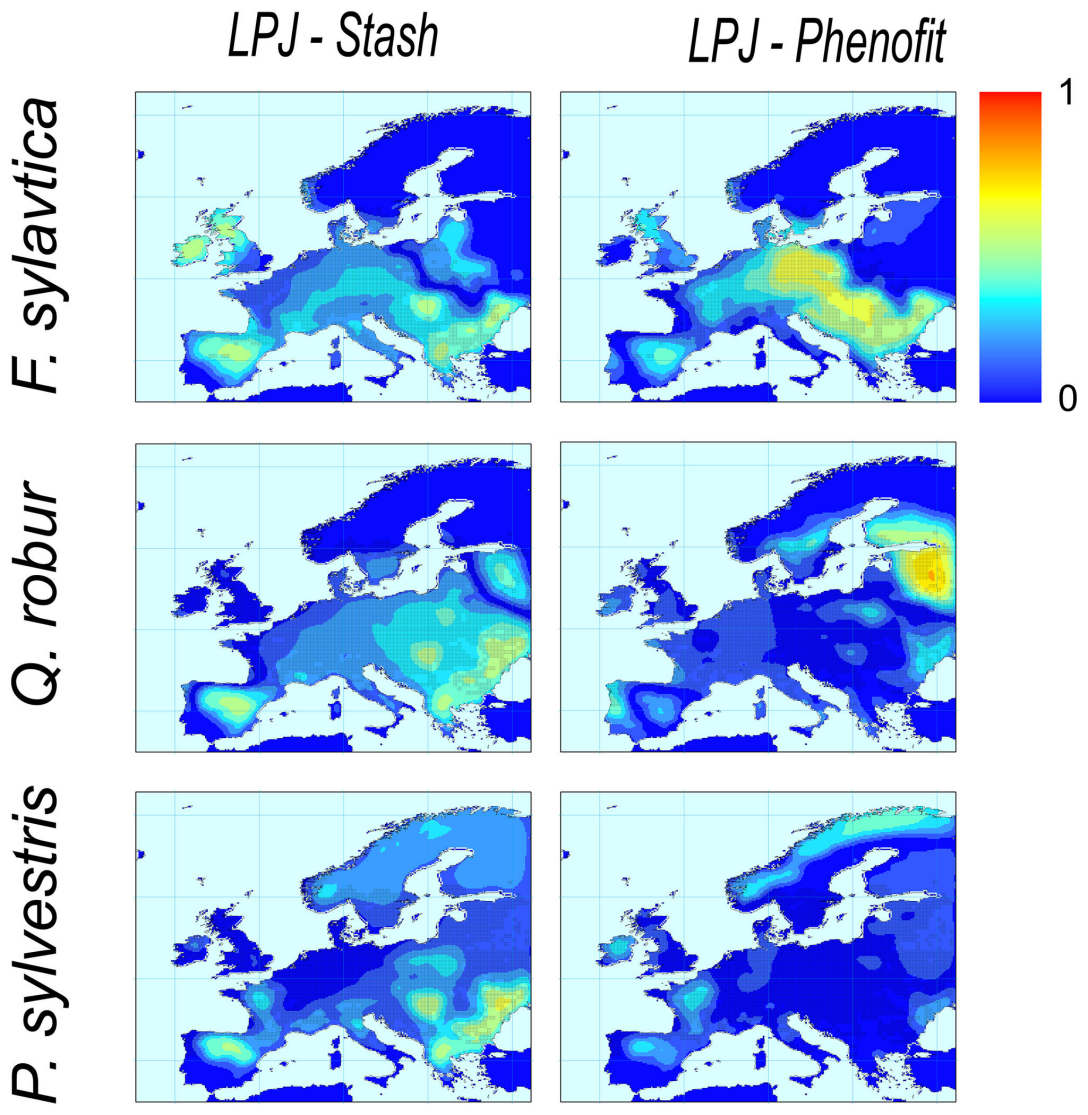
Average absolute distance between standardised model indices over the 20 monoscales (columns: LPJ-STASH; LPJ-PHENOFIT; lines: *F* *. sylvatica* ; *Q* *. robur* ; *P* *. sylvestris* ; black dots: current observed distribution).

With the exception of *F* *. sylvatica* , the absolute distance between the models’ standardised indices of species’ performance was smaller between LPJ and PHENOFIT than between LPJ and STASH (Table 2 Figure 4a). This is due to the fact that LPJ and PHENOFIT yield a high performance index (LAI for LPJ and fitness for PHENOFIT) across the species range, while STASH yields an index of occurrence probability that varies much more across the range (Figure 1). In the case of *F* *. sylvatica* , the geographical variation of the PHENOFIT index is very similar to that of STASH. The absolute distance maps (Figure 5) between LPJ and STASH allowed us to identify two main discordant areas that do not vary for the three species: northeastern Iberia Peninsula and the extended Balkanic area. This discrepancy is due to the underestimation of the effect of water stress on the species distribution in LPJ. The absolute distance between models also reveals a too-strong effect of the parameter Tcoldmins (minimum temperature sustained by the species) at the north-eastern edge of *Q* *. robur* distribution in LPJ. Therefore, model comparisons confirm the causes of projection inaccuracy previously identified with the Kappa.

**Table 2:** Absolute distance (D) and cross-correlation (CC) between model projections of the tree species present distributions ( *F* *. sylvatica* ; *Q* *. robur* ; *P* *. sylvestris* ), at the monoscale 0 (pixel by pixel comparison), D_0,_ CC_0,_ and averaged over the 20 monoscales, D_mean,_ CC _mean_ (corresponding respectively to Figures 5 & 6).

Species	Models	D_0_	D_mean_	CC_0_	CC_mean_
*F* *. sylvatica*	LPJ-STASH	0.1721	0.1797	0.6601	0.4696
	LPJ-PHENOFIT	0.1743	0.1923	0.6163	0.2151
*Q* *. robur*	LPJ-STASH	0.1650	0.1869	0.7074	0.4929
	LPJ-PHENOFIT	0.1113	0.1542	0.6768	0.2974
*P* *. sylvestris*	LPJ-STASH	0.2145	0.2007	0.6858	0.4770
	LPJ-PHENOFIT	0.0334	0.1150	0.7467	0.4838

**Figure 6:**
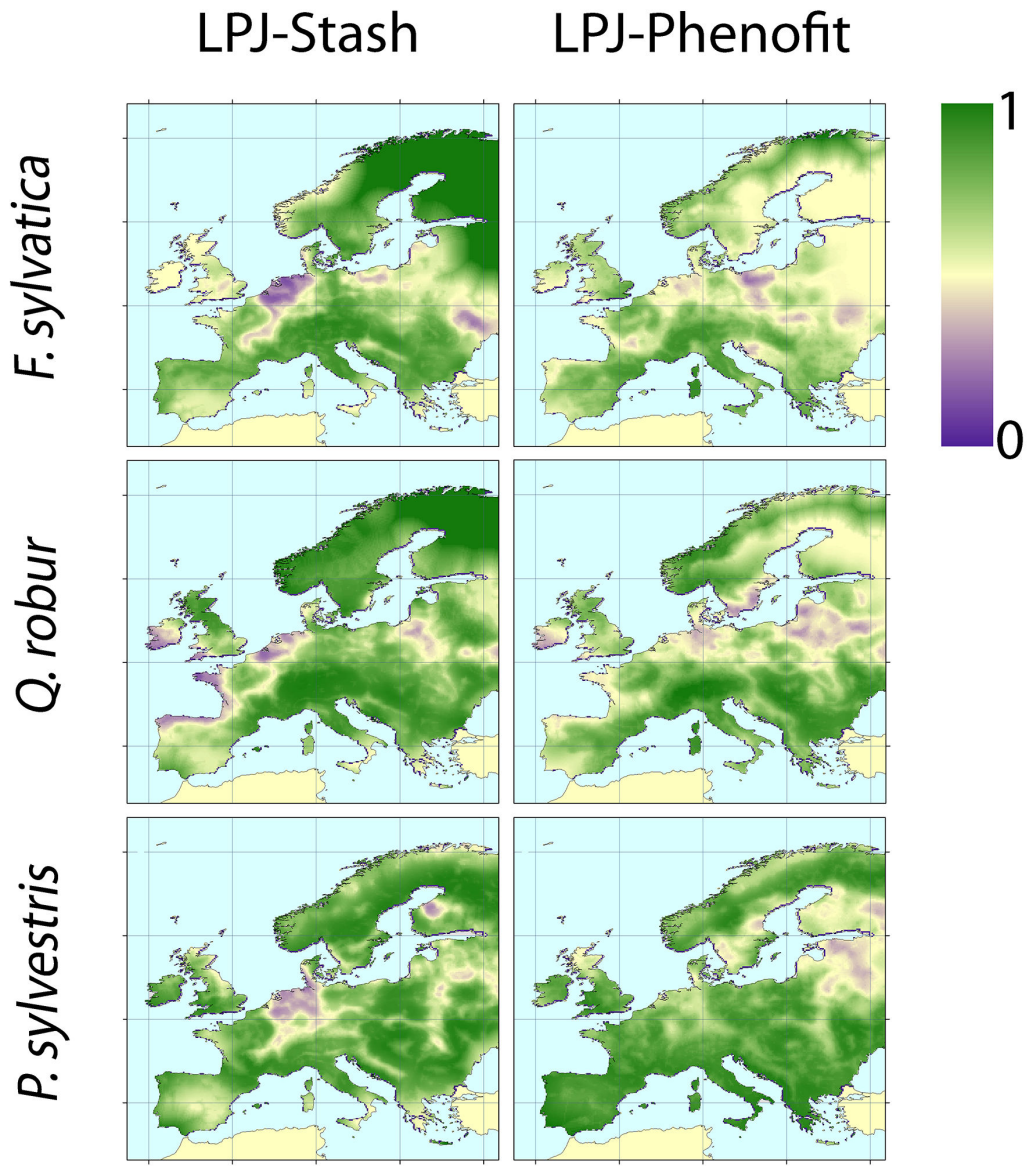
Average cross-correlation between models over the 20 monoscales (columns: LPJ-STASH; LPJ-PHENOFIT; lines: *F* *. sylvatica* ; *Q* *. robur* ; *P* *. sylvestris* ).

The cross-correlation coefficient between model projections, capturing differences in spatial structures, does not vary much across the spatial scale for the temperate broadleaved species (Figure 4b), while it increases sharply with the spatial scale for *P* *. sylvestris* . This suggests that environmental variables showing geographical patterns at global scales such as temperature are key in the distribution of this species. Correlations are generally high in most regions suggesting a predominant impact of temperature in the three models compared to water constraints (Figure 6). LPJ and STASH correlate well and more so than LPJ and PHENOFIT. The higher correlations between LPJ and STASH are the result of their more similar projected species distribution limits than a spatial correlation of their indices within the species distributions (result not shown). This again stresses the fact that bioclimatic variables seem to drive species distribution limits in LPJ while carbon and water flux drive index variation within distributions. Finally, LPJ and PHENOFIT correlate the most at high latitudes and altitudes, and in the Mediterranean area (Figure 6) and the least in the north-eastern part of Europe.

## Discussion

Our results showed that despite their totally different assumptions, the three SDMs, STASH, PHENOFIT and LPJ, provide similar and accurate projections of the current distribution of *F* *. sylvatica* *,* *Q* *. robur* and *P* *. sylvestris* current distributions. Good performance of correlative SDMs to project species current distributions, although not necessarily a good performance gage in non-analogous conditions is usually taken as grounded since the models are built on observed species distributions. Good performance of process-based models to project species current distributions is more striking since their observed distributions are normally not used to construct and parameterise the model.

Coming back to the definition of the niche by Rosenzweig (1987) as the set of traits that allows a species to survive in certain environmental conditions, the congruence observed between the process-based and the hybrid SDMs projections suggests a certain redundancy of the niche. In other words, the niche can be described by different sets of traits and/or processes, equally important. This redundancy of the niche might result from parallel evolution of all traits and processes adapting to the same environmental constraints for a particular species [56]. Indeed, why would growth be optimal if the species cannot reproduce and vice versa? Thus, it might not be necessary to have a complete description of the ecosystem in terms of traits and processes to get an accurate projection of species distributions.

Our detailed analysis of LPJ projections shows that growth processes actually do not explain species distribution limits which are almost entirely explained by the bioclimatic limits driving survival and establishment. Optimal climatic conditions for growth seem wider than the optimal conditions for reproduction and survival. LPJ has been developed initially to project biomes composition, function and distribution based on competition for light and water between different plant functional types, competition rules being driven by LAI and NPP achieved by each PFT. Processes driving survival and reproductive success have a greater importance in explaining species’ distribution limits at the European regional scale than growth processes involved in PFTs’ performances. The latter playing a larger role in explaining populations’ density patterns across a species range.

From the three models tested in this study, only PHENOFIT is able to describe species’ distribution limits solely on the basis of processes. Processes involved in resistance to abiotic stresses (ie. frost and drought), and involved in the regulation of the annual developmental cycle (*ie.* phenology), appear key in explaining species range boundaries. Still, PHENOFIT projections could be substantially improved, by representing more precisely resistance to drought; but also by representing more precisely the genetic differentiation that can arise among populations within the species range. According to our simulations, this is especially important for *P* *. sylvestris* for which the lack of observations data didn’t allow us to consider population’s phenological response heterogeneity.

The comparison of STASH, LPJ and PHENOFIT, mostly pinpoint models weaknesses, but also highlight the predominant impact of temperature on *P* *. sylvestris* distribution. Global warming may thus be a major threat to this species’ populations in Western Europe where they are already in the warmest climatic conditions they can sustain as mentioned by Reich & Oleskyn [57] and Cheaib et al. [6].

However, the main caveat of this study, like previous model intercomparison studies [6–8], is that the estimation of the accuracy of each model relies upon comparison of model projections with species presence and absence records. Each of the considered vegetation model selected in this study, projects the potential distribution of a species (even if correlative models are fitted on the species realised distribution) without considering factors such as dispersal abilities, complex biotic interactions or human activities which would explain the species present distribution. In addition, one has to note that the few databases of species distribution show major discrepancies even for common and widely distributed species such as *F* *. sylvatica* (Chuine et al., in prep). Therefore, the estimation of the model projections accuracy is highly dependent upon the reference database.

Our results also show that process-based SDMs can provide nearly as accurate projections as correlative SDMs in for the current species’ distributions. This suggests that their projections for the future may be more accurate than that of correlative SDMs because they are thought to be more robust (yet not demonstrated to our knowledge so far). Still, process-based SDMs have to achieve higher performance at broader spatial scales, which could be met by a better representation of species resistance to drought which is one of the most important weaknesses of current models. Recent advances in our understanding of the relationship between drought, plant water potential, and the different strategies of resistance to embolism and plant mortality [58–60] might allow significant improvements for models in the near future. We should also improve our representation of non-environmental factors, such as the genetic differentiation within the species range for the traits and processes, which are crucial in defining the species niche, as well as the way we handle history and human activities. Thus, considering the current caveats of the different kinds of available SDMs, our results advocate for ensemble models projections to produce reliable scenarios of species distribution change for the future [7].

## Conclusions

Our results highlight the need for model comparisons to provide more robust projections of species range shifts in the near future. Such comparison is a first step to pinpoint model weaknesses and to suggest improvement paths for failing components. But it also strongly advocates for the development of consensual methods to combine SDMs projections and uncertainties such as [7]. We show that process-based SDMs can perform almost as well as correlative SDMs despite the fact they are not parameterised on current observed species distributions, and that their prediction accuracy could be improved by integrating a more realistic representation of the species resistance to water stress. Our results also suggest that traits and processes responsible for the species distribution limits are rather those driving survival and reproductive success than those driving growth.

## Supporting Information

Appendix S1 Model description & parameterization. (DOC)

Table S1 Model parameter values. (DOC)

Figure S1 Current observed distribution of the tree species. (DOC)

Figure S2 Spatial distribution of the three monoscales kappa values of the current projection of *Pinus* *sylvestris* distribution by the model PHENOFIT. (DOC)

Figure S3 Spatial distribution of the five bioclimatic variables computed by STASH for the current period (1981-2000). (DOC)

References S1 (DOC)
