# Supplementary material for: How Can Model Comparison Help Improving Species Distribution Models?
Source: PLoS One. 2013 Jul 9;8(7):e68823. doi: 10.1371/journal.pone.0068823 (PMC3706317; doi:10.1371/journal.pone.0068823)
Supplement: Appendix S1 — (DOC) [file pone.0068823.s001.doc]

Appendix S1

**Model description**

STASH

STASH is a bioclimatic model that simulates the biogeography of individual plant species at the continental scale . This model estimates the bioclimatic envelope of the considered species by comparing its native distribution with corresponding gridded bioclimatic data. The bioclimatic envelope consists of a minimum set of physiologically constraining environmental parameters limits to plant growth and regeneration, i.e. minimum mean temperature of the coldest month (Tcoldmin), maximum mean temperature of the coldest month (Tcoldmax), minimum mean temperature of the warmest month (Twarmmin), mimnimum mean accumulated growing degree days above 5°C (GDD5min), minimum mean GDD5 requirement for budburst with no chilling (GDD5b) and a maximum mean drought index (DI). These parameters are assumed to be associated to physiological mechanisms and therefore to control species' distribution limits.

Some of these parameters act as on-off switches on the presence index calculated by STASH. For instance, if the mean temperature of the coldest month of a grid cell lies outside the interval defined by the species bioclimatic envelope, then the species is excluded from this grid cell. The other parameters act as multipliers of the presence index (see for further details).

STASH requires monthly mean values of temperature, precipitation and percentage of cloudiness, calculated over a chosen time period, in order to generate bioclimatic parameters. To define the bioclimatic envelope, it requires also a distribution map of the considered species, corresponding to the selected period.

STASH has been widely used to estimate the potential distribution of many tree species under past, current and future climatic conditions .

LPJ

LPJ is a generalized ecosystem model that combines mechanistic representations of plant physiological and biogeochemical processes with explicit formulations of the dynamic processes controlling vegetation structure, such as mortality, plant establishment, competition inter and/or intra species/Plant Functional Types (PFTs) (e.g. temperate broadleaf evergreen tree, C3 grass/herb). The model simulates the growth of individual trees on a number of replicate patches, corresponding approximately in size to the area of influence that one large adult tree has on its neighbours. Herbaceous vegetation is also represented, but individuals are not distinguished. Tree height and diameter growth are regulated by carbon allocation, conversion of sapwood to heartwood, and a set of prescribed allometric relationships. Litter and soil organic matter carbon (divided in three pools) dynamics follow first-order kinetics and are sensitive to temperature and soil water content. Leaf and root turnover and plant mortality replenish the litter pool. While this model includes a large amount of plant physiology, four bioclimatic limits are used as surrogate for poorly understood physiological constraints on survival and establishment : minimum GDD5 for establishment (GDD5mine); minimum temperature of the coldest month for survival and establishment (respectively Tcoldmins and Tcoldmine); maximum temperature of the coldest month for establishment (Tcoldmaxe) (see Sitch for further details). The version used in this study includes representations of soil hydrology, snow-pack dynamics and soil–vegetation–atmosphere exchange of water, as documented by Gerten *et.al.* .

Climatic factors, expressed as monthly temperature, precipitation and cloudiness, as well as a prescribed disturbance regime which can be used to represent land management, directly influence vegetation distribution and dynamics. Output variables of LPJ are net primary production (NPP), leaf area index (LAI) and biomass. Each species is defined by a set of parameters describing plant physionomy, allometry, physiology, phenology and bioclimatic limits. The species parameter’ sets used in the present study were based on the parameter sets of the corresponding PFTs defined in the global version of LPJ (Temperate Broadleaved Summergreen for *F. sylvatica* and *Q. robur*; Boreal Needleleaved Evergreen for *P. sylvestris*). However species-specific values were used when available.

In this study LPJ predicts potential distribution of the species described by climate and intraspecific interactions within an ecosystem. Human land use is not included in the simulations. However, the effects of land use on potential vegetation are simulated as mean stochastic disturbance regime of 40 years by removing the biomass present on the randomly selected patches and attributing it to the litter pool.

The model has been validated by several previous studies at the PFT level [8-10];. The modelling of ecosystem processes has also been validated with respect to seasonal and interannual variation in carbon and water vapour fluxes , (Koca *et.al.*, in prep.).

PHENOFIT

PHENOFIT is a process-based model describing tree species potential distribution and is based on the concept of fitness. In its present version it estimates the fitness of an average individual of a particular species in response to climatic and environmental conditions representing an estimate of its probability of presence. The model relies on the assumption that species adaptation to abiotic conditions is tightly related to its capacity to synchronise its annual life cycle with seasonal climatic variations impacting directly its survival and reproductive success. Annual presence probability of the considered species is the product of 1) its probability to survive to climatic stress (frost and drought) until the following reproductive season (survival index) and 2) its probability to produce viable seeds before the end of its current annual cycle (reproductive success). PHENOFIT is composed of several process-based sub-models of phenology (leaf unfolding, flowering, fruit maturation, leaf senescence), frost injury on leaves, flowers and fruits. The version used in this study does not mechanistically describe the drought resistance of each species, and survival to drought is described by the amount of annual precipitation encountered over the species range.

PHENOFIT requires monthly mean values of precipitation and daily values of temperatures, calculated over a chosen time period, in order to estimate the fitness of a given species across the considered window.

This model has been used at the continental scale and validated for a dozen of American tree species .

**Model parameterization**

STASH

Atlas Flora Europea current observed species distribution maps completed by Laurent *et al.* were used with STASH to define the species bioclimatic envelopes. These maps compile species presence records from the second half of the 20^th^ century and are assumed to reflect the most accurate approximation of the species distribution available at the European scale, resulting from last normal climatic conditions period (1931-1960) . These maps were standardised to a 0.5° x 0.5° grid at the European window. However to ease their use with the climatic data set, these maps were downscaled to a 10’x10’ resolution by attributing the value of the 0.5° cells to the set of corresponding 10’ cells.

We used a GIS to compare species distribution maps to spatialized bioclimatic parameters (See Appendix S5) and define their initial bioclimatic envelopes’ boundaries. To avoid overestimation of the niche amplitude, we neglected outliers outside the 2.5-97.5 percentiles.

Values associated to minimum GDD5 for budburst with no chilling requirement were fixed to those proposed by Sykes *et.al.* based on previous experimental work from Murray *et.al.* .

LPJ

Assignment of species-specific parameters for the tree three species of interest followed the approach of Hickler *et al.* .Concerning parameters directly influencing ecophysiology and life history generic values of the corresponding plant functional types (climatic range, leaf type, phenology...see appendix S2) were used. Yet, specific bioclimatic limits for establishment and/or survival obtained with STASH were used when available. A prescribed perturbation rate of 40 years was applied, as recognized by the CORINE code corresponding to a moderate exploitation of the habitat for firewood, construction wood, utility wood etc.

PHENOFIT

Parameters of the phenological sub-models of PHENOFIT were fitted using field phenological observations of leaf unfolding, flowering, fruit maturation and senescence from different populations of each species provided by the French Phenologial Database (Observatoire des Saisons, GDR2968, [www.gdr2968.cnrs.fr](http://www.gdr2968.cnrs.fr/) ). Leaf unfolding and senescence dates were collected over the period 1997-2006, flowering dates were collected over the period 2006-2008. Fruit maturation dates were derived from seed collection campaign of the Seed Service of the French Forest Service (ONF, Sécherie de la Joux) since 1990. Observations from the different sites where aggregated following the Inventaire Forestier National (IFN) description of the provenance regions for each species. Each phenological models was parameterized for each provenance region using the phenological time series and daily complete temperature series from the nearest meteorological stations provided by MeteoFrance. The leaf unfolding date and flowering date model used was the Unichill model . The fruit maturation model is the original fruit maturation sub-model of PHENOFIT . The leaf senescence date is computed as a linear function of latitude fitted on the observed date of coloration of leaves at the latitudinal boundaries of the focal species distribution .

Parameterized leaf unfolding models for *P. sylvestris* were not satisfactory. We therefore used the parameters previously fitted by Kramer with German provenances for our study. Resistance to drought was derived from precipitation limits known for these species by the French National Forest Inventory (http://www.ifn.fr/spip/). Lethal temperatures were taken from Sakai & Weiser . Frost hardiness model parameters were set to the original values except the minimum and maximum frost resistance parameters that were set according to the literature (Appendix S1). Resistance to drought and frost parameters were unique for the each species contrary to the phenological model parameters.

Simulations are conducted with each provenance region parameter set and then aggregated using the maximum fitness value obtained at each grid point.
