# Supplementary material for: How Can Model Comparison Help Improving Species Distribution Models?
Source: PLoS One. 2013 Jul 9;8(7):e68823. doi: 10.1371/journal.pone.0068823 (PMC3706317; doi:10.1371/journal.pone.0068823)
Supplement: Table S1 — (DOC) [file pone.0068823.s002.doc]

Table S1: Model parameter values

|  | ***F. sylvatica*** | | | | | | | | ***Q. robur*** | | | | | | | | | | ***P. sylvestris*** | |
| --- | --- | --- | --- | --- | --- | --- | --- | --- | --- | --- | --- | --- | --- | --- | --- | --- | --- | --- | --- | --- |
| **STASH** |  | | | | | | | |  | | | | | | | | | |  | |
| Tcoldmin | -3.5 | | | | | | | | -6.2 | | | | | | | | | | -16.2 | |
| Tcoldmax | 6.1 | | | | | | | | 10 | | | | | | | | | | 4.2 | |
| Twarmmin | 12.9 | | | | | | | | 5 | | | | | | | | | | 9 | |
| GDD5min  GDD5b | 820  1150 | | | | | | | | 653  100 | | | | | | | | | | 238  100 | |
| DImax | 0.317 | | | | | | | | 0.341 | | | | | | | | | | 0.230 | |
| **LPJ** |  | | | | | | | |  | | | | | | | | | |  | |
| **Bioclimatic limits** |  | | | | | | | |  | | | | | | | | | |  | |
| Tcoldmins | -4 | | | | | | | | -7 | | | | | | | | | | -35 | |
| Tcoldmine | -4 | | | | | | | | -7 | | | | | | | | | | -30 | |
| Tcoldmaxe | 6.1 | | | | | | | | 5 | | | | | | | | | | 3 | |
| GDD5mine | 1000 | | | | | | | | 900 | | | | | | | | | | 400 | |
| **Ecophysiology** |  | | | | | | | |  | | | | | | | | | |  | |
| Roots distribution  (upper/lower soil layer) | 0.67/0.33 | | | | | | | | 0.67/0.33 | | | | | | | | | | 0.67/0.33 | |
| Leaf Phenology | Summergreen | | | | | | | | Summergreen | | | | | | | | | | Evergreen | |
| Leaf turnover rate (year^-1^) | 1 | | | | | | | | 1 | | | | | | | | | | 0.33 | |
| SLA (cm^2^.[gC]^-1^) | 273 | | | | | | | | 273 | | | | | | | | | | 93 | |
| Climate zone | Temperate | | | | | | | | Temperate | | | | | | | | | | Boreal | |
| Optimal temperature range for photosynthesis (°C) | 15-25 | | | | | | | | 15-25 | | | | | | | | | | 10-25 | |
| Max establishment (saplings.ha^-1^.yr^-1^) | 10 | | | | | | | | 10 | | | | | | | | | | 10 | |
| Max non-stressed longevity (yr) | 200 | | | | | | | | 200 | | | | | | | | | | 300 | |
| **Phenofit** |  | | | | | | | |  | | | | | | | | | |  | |
|  | Prov201 | | Prov403 | | Prov602 | | Prov751 | |  | | Prov100 | | Prov201 | | Prov361 | |  | | Prov1 | |
| **Leafing** | |  | |  | |  | |  | |  | |  | |  | |  | |  | |  |
| a | | 0.54 | | 3.65 | | 1.04 | | 1.13 | |  | | 1,14 | | 0,56 | | 0,96 | |  | | 0,06 |
| b | | -19.52 | | -22.01 | | -26.68 | | -28.43 | |  | | -22,04 | | -3,52 | | -21,51 | |  | | 1 |
| c | | -19.86 | | 11 | | -6.22 | | -13.57 | |  | | -1,71 | | 0,18 | | -4,2 | |  | | 6 |
| d | | -40 | | -0.1 | | -40 | | -7.13 | |  | | -40 | | -40 | | -0,35 | |  | | -0,11 |
| e | | 8.4 | | 2.73 | | 8.55 | | 9.94 | |  | | 6,72 | | 9,92 | | 6,11 | |  | | 37 |
| C* | | 202.51 | | 12.75 | | 218.85 | | 136.43 | |  | | 182,39 | | 4,82 | | 210,79 | |  | | 85 |
| F* | | 9.5 | | 121 | | 4.2 | | 20.5 | |  | | 19,9 | | 31 | | 10,6 | |  | | 2,4 |
| **Flowering** | |  | |  | |  | |  | |  | |  | |  | |  | |  | |  |
| F** | | 18.5 | | 129.5 | | 12.7 | | 29 | |  | | 25,39 | | 35,8 | | 15,92 | |  | | 2,2 |
| **Fruiting** | |  | |  | |  | |  | |  | |  | |  | |  | |  | |  |
| aa | | -16.74  14.72  9.26  5.002  104.5  50.14  0.4  // | | | | | | | |  | | -10,15 | | -0,25 | | -3,97 | |  | | // |
| bb | |  |  |  |  |  |  |  |  |  | | 13,96 | | 18,1 | | 9,46 | |  | | // |
| Fcrit | |  |  |  |  |  |  |  |  |  | | 5,97 | | 30,28 | | 120,82 | |  | | 500 |
| Top | |  |  |  |  |  |  |  |  |  | | 5 | | 6,56 | | 19,77 | |  | | // |
| matmoy | |  |  |  |  |  |  |  |  |  | | 136,34 | | 102,95 | | 47,77 | |  | | // |
| sigma | |  |  |  |  |  |  |  |  |  | | 37,44 | | 46,34 | | 28,21 | |  | | 57 |
| pfe50 | |  |  |  |  |  |  |  |  |  | | 0,4 | | 0,4 | | 0,4 | |  | | 0,4 |
| Tb | |  |  |  |  |  |  |  |  |  | | // | | // | | // | |  | | 5 |
| **Frost injury** | |  | |  | |  | |  | |  | |  | |  | |  | |  | |  |
| Frmax1 | | -5  -20  -4  -2.5  10  -16  -13  -12 | | | | | | | |  | | -12  -50  -7  -7  10  -16  -41  -60 | | | | | |  | | -10 |
| Frmax2 | |  |  |  |  |  |  |  |  |  | |  |  |  |  |  |  |  | | -50 |
| Flmin | |  |  |  |  |  |  |  |  |  | |  |  |  |  |  |  |  | | -5 |
| Ffmin | |  |  |  |  |  |  |  |  |  | |  |  |  |  |  |  |  | | -10 |
| T1 | |  |  |  |  |  |  |  |  |  | |  |  |  |  |  |  |  | | 10 |
| T2 | |  |  |  |  |  |  |  |  |  | |  |  |  |  |  |  |  | | -16 |
| Ftlmax | |  |  |  |  |  |  |  |  |  | |  |  |  |  |  |  |  | | -47 |
| Ftfmax | |  |  |  |  |  |  |  |  |  | |  |  |  |  |  |  |  | | -47 |
| Fplmax | | -7  -6  10 | | | | | | | |  | | -21  -20  10  16 | | | | | |  | | -18,5 |
| Fpfmax | |  |  |  |  |  |  |  |  |  | |  |  |  |  |  |  |  | | -18,5 |
| NL1 | |  |  |  |  |  |  |  |  |  | |  |  |  |  |  |  |  | | 10 |
| NL2 | | 16 | | | | | | | |  | |  |  |  |  |  |  |  | | 16 |
| **Precip Limits** | |  | |  | |  | |  | |  | |  | |  | |  | |  | |  |
| PPmin | | 730  1440 | | | | | | | |  | | 600  2030 | | | | | |  | | 560 |
| PPmax | |  |  |  |  |  |  |  |  |  | |  |  |  |  |  |  |  | | 3200 |
