# Supplementary figures and images for: How Can Model Comparison Help Improving Species Distribution Models?
Source: PLoS One. 2013 Jul 9;8(7):e68823. doi: 10.1371/journal.pone.0068823 (PMC3706317; doi:10.1371/journal.pone.0068823)

Figure S1: Current observed distribution of the tree species


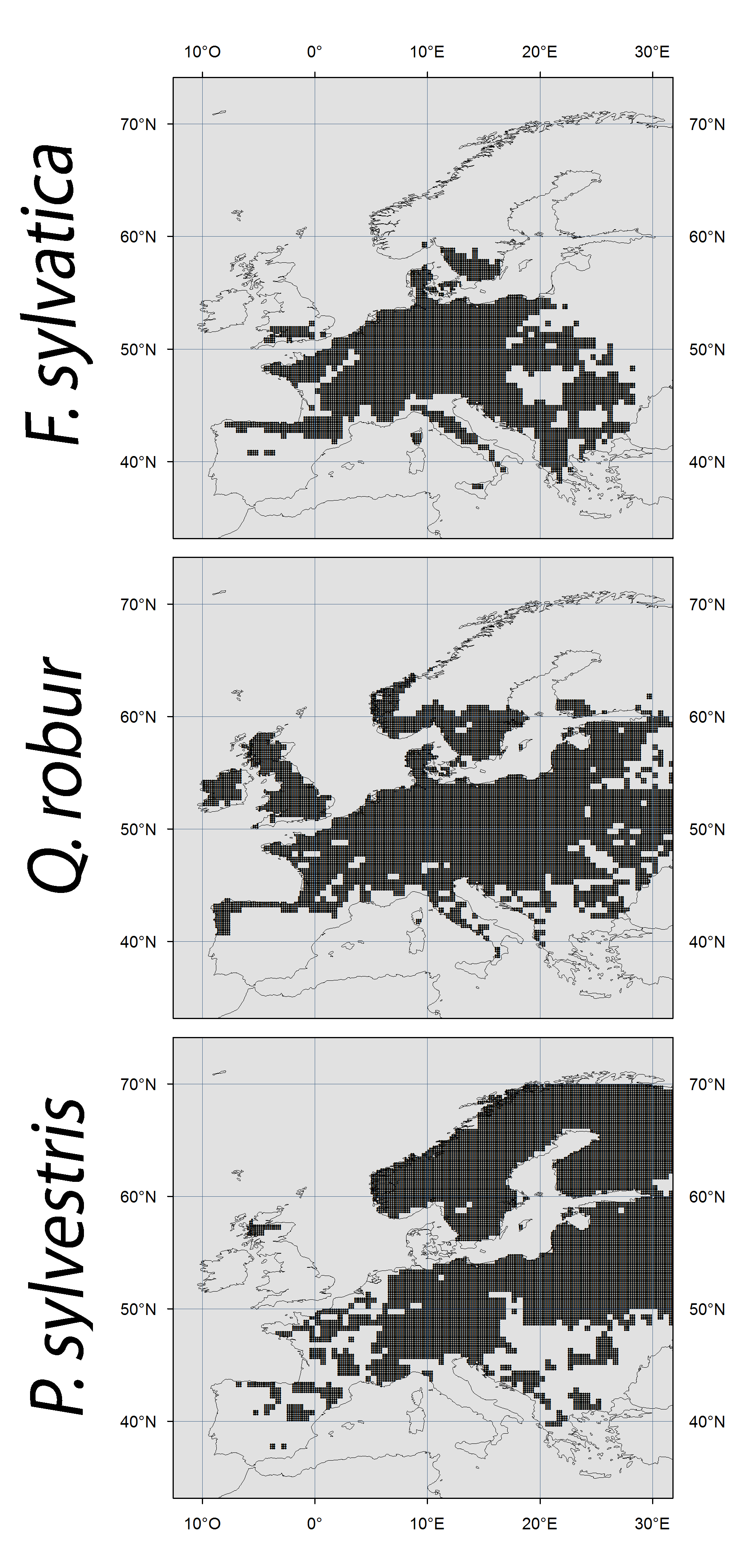

Supplement: Figure S1 — (DOC) [file pone.0068823.s003.doc]
