# Supplementary material for: How Can Model Comparison Help Improving Species Distribution Models?
Source: PLoS One. 2013 Jul 9;8(7):e68823. doi: 10.1371/journal.pone.0068823 (PMC3706317; doi:10.1371/journal.pone.0068823)

Figure S2: Spatial distribution of the three monoscales kappa values of the current projection of *Pinus sylvestris* distribution by the model PHENOFIT.


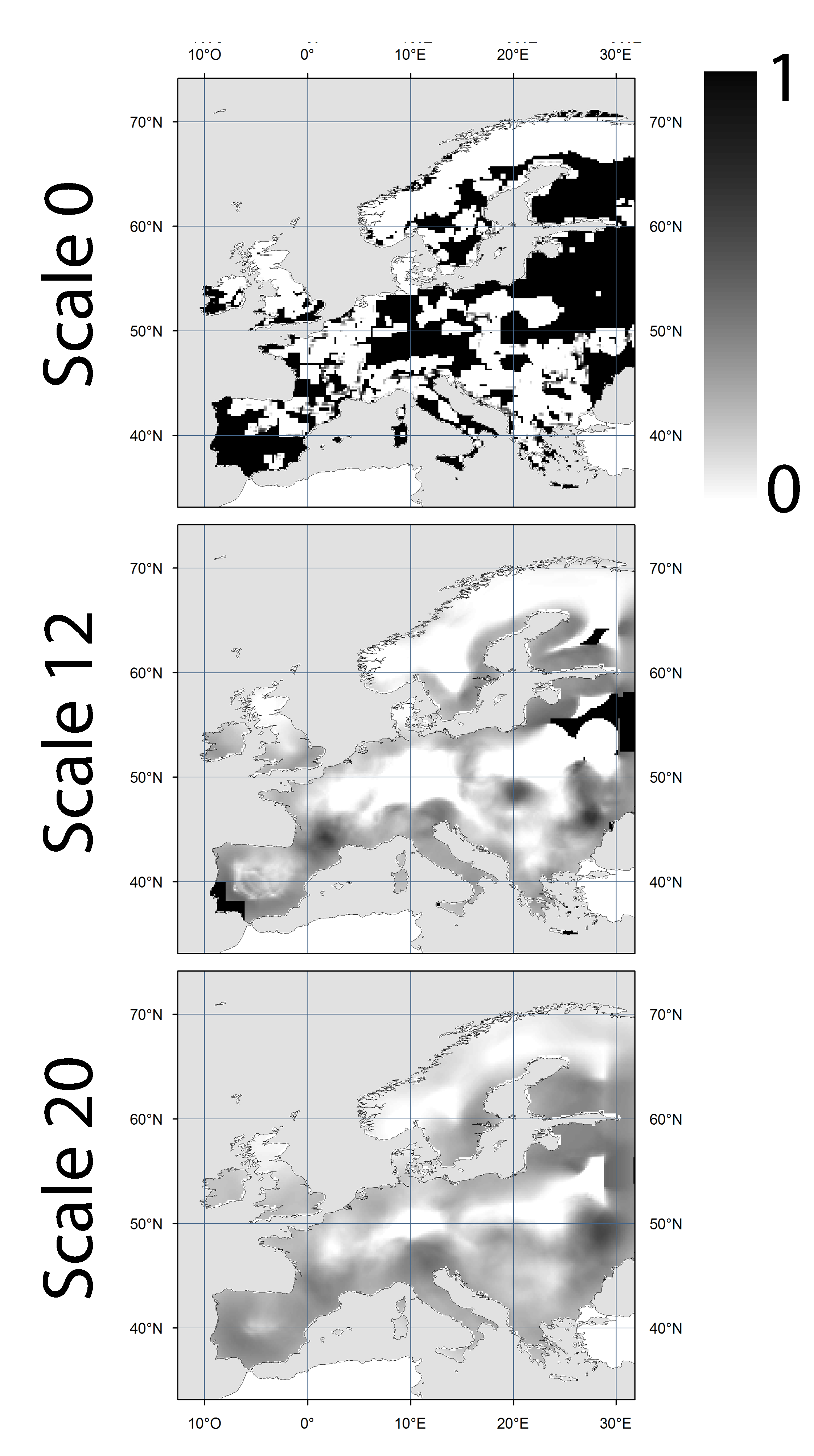

Supplement: Figure S2 — (DOC) [file pone.0068823.s004.doc]
