# Supplementary material for: How Can Model Comparison Help Improving Species Distribution Models?
Source: PLoS One. 2013 Jul 9;8(7):e68823. doi: 10.1371/journal.pone.0068823 (PMC3706317; doi:10.1371/journal.pone.0068823)
Supplement: Figure S3 — (DOC) [file pone.0068823.s005.doc]

Figure S3: Spatial distribution of the five bioclimatic variables computed by STASH for the current period (1981-2000).

**
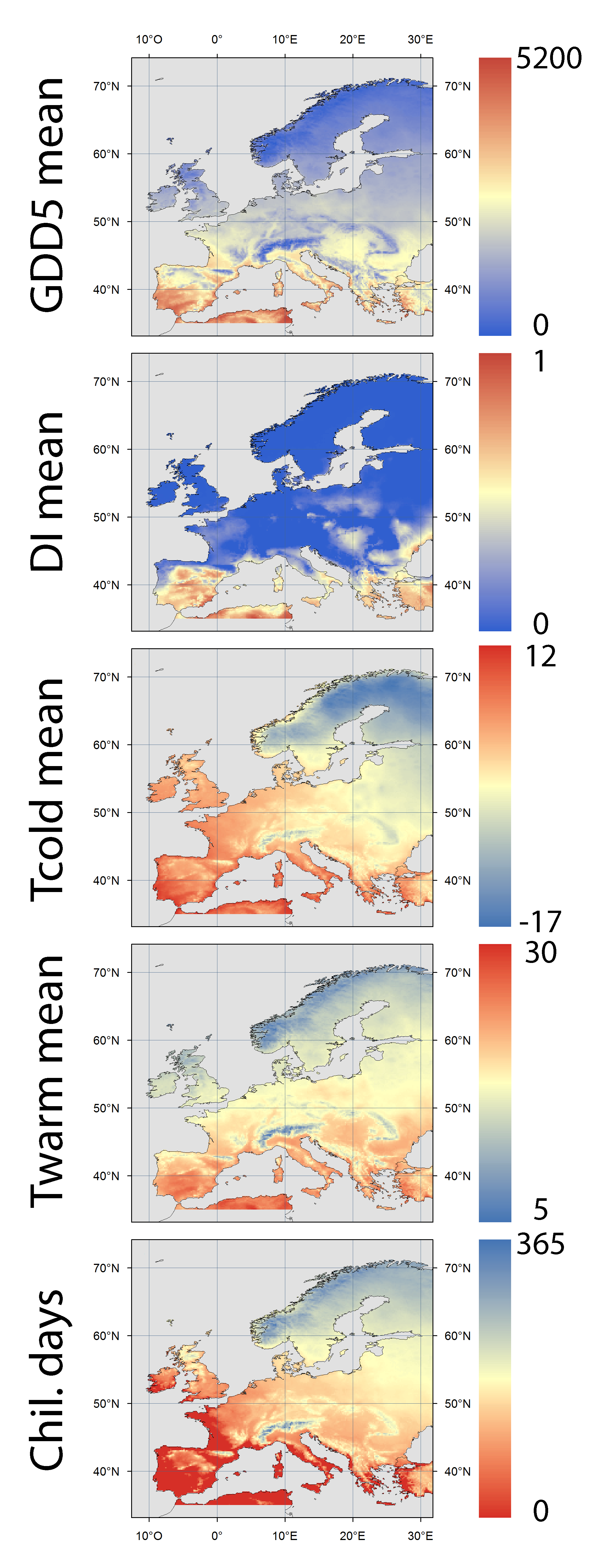
**
