# Supplementary material for: How Can Model Comparison Help Improving Species Distribution Models?
Source: PLoS One. 2013 Jul 9;8(7):e68823. doi: 10.1371/journal.pone.0068823 (PMC3706317; doi:10.1371/journal.pone.0068823)
Supplement: References S1 — (DOC) [file pone.0068823.s006.doc]

References S1:

1. Sykes MT, Prentice IC, Cramer W (1996) A bioclimatic model for the potential distributions of north European tree species under present and future climates. Journal of Biogeography 23: 203-233.

2. Giesecke T, Hickler T, Kunkel T, Sykes MT, Bradshaw RHW (2007) Towards an understanding of the Holocene distribution of Fagus sylvatica L. Journal of Biogeography 34: 118-131.

3. Walther GR, Gritti ES, Berger S, Hickler T, Tang ZY, et al. (2007) Palms tracking climate change. Global Ecology and Biogeography 16: 801-809.

4. Sitch S, Smith B, Prentice IC, Arneth A, Bondeau A, et al. (2003) Evaluation of ecosystem dynamics, plant geography and terrestrial carbon cycling in the LPJ dynamic global vegetation model. Global Change Biology 9: 161-185.

5. Prentice IC, Cramer W, Harrison SP, Leemans R, Monserud RA, et al. (1992) A global biome model based on plant physiology and dominance, soil properties and climate. Journal of Biogeography 19: 117-134.

6. Sykes MT, Prentice IC, Cramer W (1996) A bioclimatic model for the potential distributions of north European tree species under present and future climates. Journal of Biogeography 23: 203-233.

7. Gerten D, Schaphoff S, Haberlandt U, Lucht W, Sitch S (2004) Terrestrial vegetation and water balance - hydrological evaluation of a dynamic global vegetation model. Journal of Hydrology 286: 249-270.

8. Smith B, Prentice IC, Sykes MT (2001) Representation of vegetation dynamics in the modelling of terrestrial ecosystems: comparing two contrasting approaches within European climate space. Global Ecology and Biogeography 10: 621-637.

9. Morales P, Sykes MT, Prentice IC, Smith P, Smith B, et al. (2005) Comparing and evaluating process-based ecosystem model predictions of carbon and water fluxes in major European forest biomes. Global Change Biology 11: 2211-2233.

10. Chuine I, Beaubien EG (2001) Phenology is a major determinant of tree species range. Ecology Letters 4: 500-510.

11. Morin X, Augspurger C, Chuine I (2007) Process-based modeling of species' distributions: What limits temperate tree species' range boundaries? Ecology 88: 2280-2291.

12. Morin X, Thuiller W (2009) Comparing niche- and process-based models to reduce prediction uncertainty in species range shifts under climate change. Ecology 90: 1301-1313.

13. Morin X, Viner D, Chuine I (2008) Tree species range shifts at a continental scale: new predictive insights from a process-based model. Journal of Ecology 96: 784-794.

14. Tutin TG, Heywood VH, Burges NA, Moore DM (1964-85) Flora Europaeae. Cambridge: Cambridge University Press.

15. Laurent JM, Bar-Hen A, Francois L, Ghislain M, Cheddadi R (2004) Refining vegetation simulation models: From plant functional types to bioclimatic affinity groups of plants. Journal of Vegetation Science 15: 739-746.

16. Ohlemuller R, Gritti ES, Sykes MT, Thomas CD (2006) Towards European climate risk surfaces: the extent and distribution of analogous and non-analogous climates 1931-2100. Global Ecology and Biogeography 15: 395-405.

17. Murray MB, Cannell MGR, Smith RI (1989) Date of Budburst of Fifteen Tree Species in Britain Following Climatic Warming. Journal of Applied Ecology 26: 693-700.

18. Hickler T, Smith B, Sykes MT, Davis MB, Sugita S, et al. (2004) Using a generalized vegetation model to simulate vegetation dynamics in northeastern USA. Ecology 85: 519-530.

19. Romão C (1996) Manuel d’interprétation des habitats de l’Union européenne. Version EUR 15. Bruxelles: Commission européenne - DG XI. 109 p.

20. Chuine I, Cour P, Rousseau DD (1999) Selecting models to predict the timing of flowering of temperate trees: implications for tree phenology modelling. Plant Cell and Environment 22: 1-13.

21. Lamb GN (1915) A calendar of the leafing, flowering and seeding of the common trees of the eastern United States. Monthly Weather Review Supplement: 5-19.

22. Kramer K (1994) A modelling analysis on the effects of climatic

warming on the probability of spring frost damage to tree species

in The Netherlands and Germany. Plant, Cell and Environment

17: 367-377.

23. Sakai A, Weiser CJ (1973) FREEZING RESISTANCE OF TREES IN NORTH-AMERICA WITH REFERENCE TO TREE REGIONS. Ecology 54: 118-126.

24. Leinonen I (1996) A simulation model for the annual frost hardiness and freeze damage of Scots pine. Annals of Botany 78: 687-693.
